# Supplementary material for: Predicting visual function by interpreting a neuronal wiring diagram
Source: Nature. 2024 Oct 2;634(8032):113–23. doi: 10.1038/s41586-024-07953-5 (PMC11446822; doi:10.1038/s41586-024-07953-5)
Supplement: Supplementary file 6 — CRF and ERF predictions for individual TmY4 and TmY9 cells. Analogous to Supplementary Data 3, but for TmY target types. Shown are the top four monosynaptic pathways, the strongest pathway passing through each of the top ten intermediary types (ranking from Extended Data Fig. 7), and the trisynaptic pathway Tm1–TmY–Dm3–TmY (see the section entitled Prediction of spatial normalization). [file 41586_2024_7953_MOESM6_ESM.zip › DataS4/TmY9q/720575940623077731.pdf]

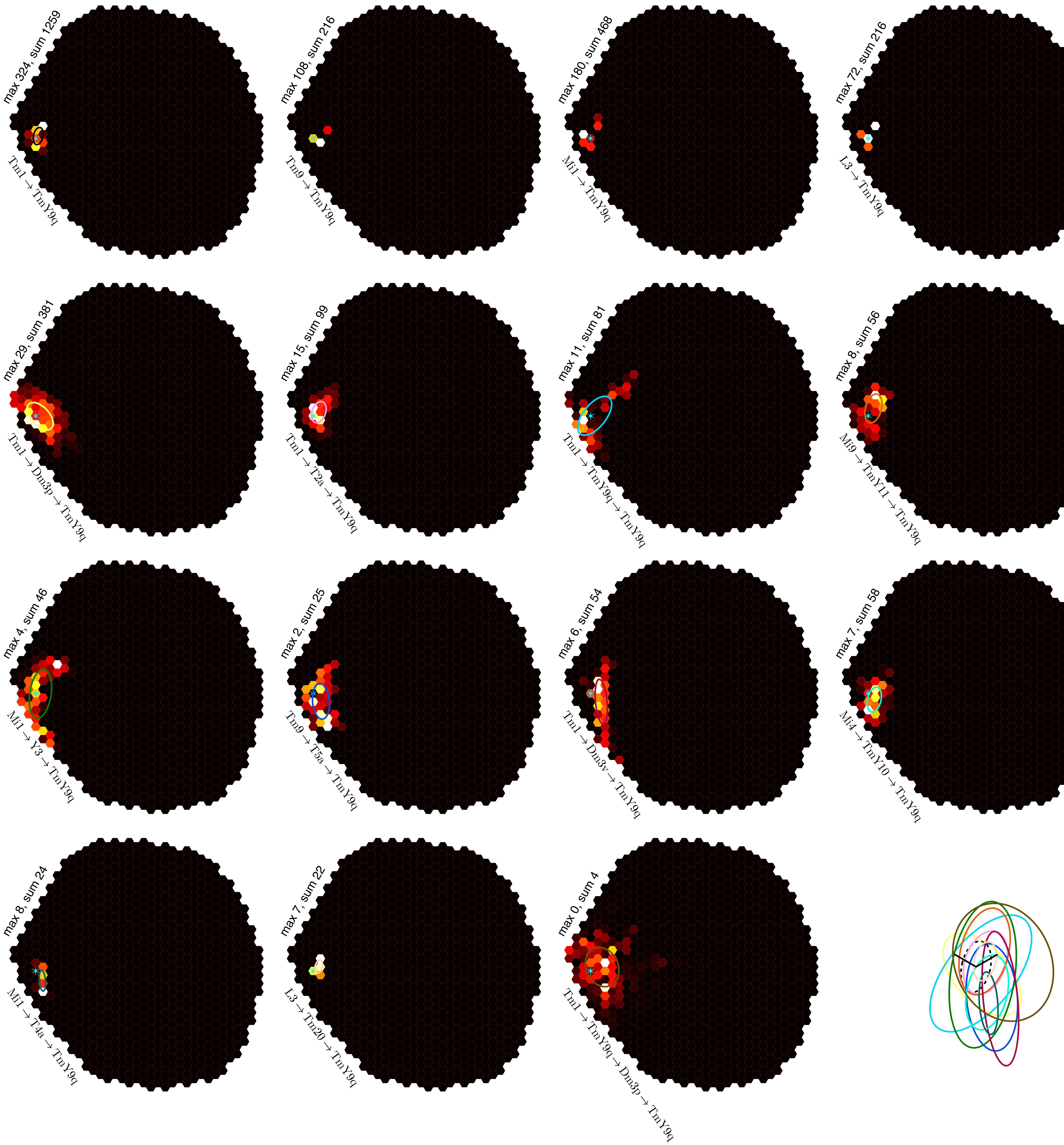
max 324, sum 1259
Tm1 → TmY9q
max 108, sum 216
Tm9 → TmY9q
max 180, sum 468
Mi1 → TmY9q
max 72, sum 216
L3 → TmY9q
max 29, sum 381
Tm1 → Dm3p → TmY9q
max 15, sum 99
Tm1 → T2a → TmY9q
max 11, sum 81
Tm1 → TmY9q → TmY9q
max 8, sum 56
Mi9 → TmY11 → TmY9q
max 4, sum 46
Mi1 → Y3 → TmY9q
max 2, sum 25
Tm9 → T5a → TmY9q
max 6, sum 54
Tm1 → Dm3v → TmY9q
max 7, sum 58
Mi4 → TmY10 → TmY9q
max 8, sum 24
Mi1 → T4a → TmY9q
max 7, sum 22
L3 → Tm20 → TmY9q
max 0, sum 4
Tm1 → TmY9q → Dm3p → TmY9q
